# Supplementary material for: Genome-wide CRISPR Screens in T Helper Cells Reveal Pervasive Crosstalk between Activation and Differentiation
Source: Cell. 2019 Feb 7;176(4):882–896.e18. doi: 10.1016/j.cell.2018.11.044 (PMC6370901; doi:10.1016/j.cell.2018.11.044)
Supplement: Data S2. Processed Data from All the Steps of the Analysis, Related to Figure 1 [file mmc2.zip › supplemental data/motif analysis/Stat6_SRX021632_homer/homerResults/motif5.similar.html]

motif5

## Information for motif5

C
A
T
G
G
A
C
T
C
G
A
T
A
C
T
G
G
C
A
T
C
T
A
G
A
C
T
G
A
G
C
T
G
A
C
T
C
G
A
T
  
Reverse Opposite:  

G
C
T
A
C
T
G
A
T
C
G
A
A
G
T
C
A
G
T
C
C
G
T
A
A
G
T
C
G
C
T
A
C
T
G
A
G
T
A
C
  

|  |  |
| --- | --- |
| p-value: | 1e-654 |
| log p-value: | -1.506e+03 |
| Information Content per bp: | 1.711 |
| Number of Target Sequences with motif | 3777.0 |
| Percentage of Target Sequences with motif | 14.94% |
| Number of Background Sequences with motif | 1333.8 |
| Percentage of Background Sequences with motif | 5.52% |
| Average Position of motif in Targets | 101.0 +/- 49.8bp |
| Average Position of motif in Background | 100.6 +/- 61.8bp |
| Strand Bias (log2 ratio + to - strand density) | -0.1 |
| Multiplicity (# of sites on avg that occur together) | 1.11 |
| Motif File: | file (matrix) reverse opposite |

### Similar de novo motifs found

|  |  |  |  |  |  |  |  |
| --- | --- | --- | --- | --- | --- | --- | --- |
| Rank | Match Score | Redundant Motif | P-value | log P-value | % of Targets | % of Background | Motif file |
| 1 | 0.978 | A C G T A C T G A G C T A C T G A C T G A C G T A C G T C G A T | 1e-517 | -1191.860295 | 7.75% | 2.08% | motif file (matrix) |
| 2 | 0.971 | G T C A T C A G T C A G G T C A C T G A T C G A A T G C A G T C G C T A T G A C G C T A T C A G | 1e-510 | -1174.835177 | 10.92% | 3.82% | motif file (matrix) |
| 3 | 0.877 | A C T G G A C T C T A G C T A G A G C T G C A T C A G T G A T C | 1e-417 | -961.441085 | 37.01% | 24.61% | motif file (matrix) |
| 4 | 0.834 | G A C T C G A T T C G A A G T C T A G C C T G A A T G C G C T A | 1e-410 | -944.877910 | 40.47% | 27.78% | motif file (matrix) |
| 5 | 0.816 | C A G T A T G C G A C T G C A T T C G A A T G C G T A C C G T A G T A C G T C A | 1e-295 | -680.395879 | 13.23% | 6.73% | motif file (matrix) |
| 6 | 0.721 | A G C T A C G T A C T G A G T C A C T G A T C G A G C T A G C T A C G T A T G C | 1e-286 | -660.148045 | 9.40% | 4.15% | motif file (matrix) |
| 7 | 0.643 | A G T C G A C T A C G T C A G T A T C G C T G A C G A T A C T G C G A T C A T G A C T G A C G T | 1e-53 | -123.195490 | 0.26% | 0.02% | motif file (matrix) |
| 8 | 0.841 | A C G T A C T G A C G T A C T G A T C G A C T G A C G T A C G T | 1e-32 | -75.823632 | 1.90% | 1.04% | motif file (matrix) |
